# Supplementary material for: Evolution of the conformational dynamics of the molecular chaperone Hsp90
Source: Nat Commun. 2024 Oct 4;15:8627. doi: 10.1038/s41467-024-52995-y (PMC11452706; doi:10.1038/s41467-024-52995-y)
Supplement: Supplementary file 1 — Supplementary Information [file 41467_2024_52995_MOESM1_ESM.pdf]

SUPPLEMENTARY INFORMATION

Evolution of the conformational dynamics of the molecular chaperone Hsp90

Stefan Riedl[1], Ecenaz Bilgen[2], Ganesh Agam[2], Viivi Hirvonen[3], Alexander Jussupow[3], Franziska Tippl[1], Maximilian Riedl[1], Andreas Maier[1], Christian F. W. Becker[4], Ville R. I. Kaila[3], Don C. Lamb[2] and Johannes Buchner[1]*

[1] Center for Protein Assemblies, Department Bioscience, School of Natural Sciences, Technical University Munich, Ernst-Otto-Fischer-Straße 8, 85748 Garching, Germany

[2] Department of Chemistry and Center for Nanoscience, Ludwig-Maximilians-Universität Munich, 81377 Munich, Germany

[3] Department of Biochemistry and Biophysics, The Arrhenius Laboratories for Natural Sciences, Stockholm University, SE-106 91, Stockholm, Sweden

[4] Institute of Biological Chemistry, Faculty of Chemistry, University of Vienna, Währinger Straße 38, 1090 Vienna, Austria

* to whom correspondence should be addressed at johannes.buchner@tum.de

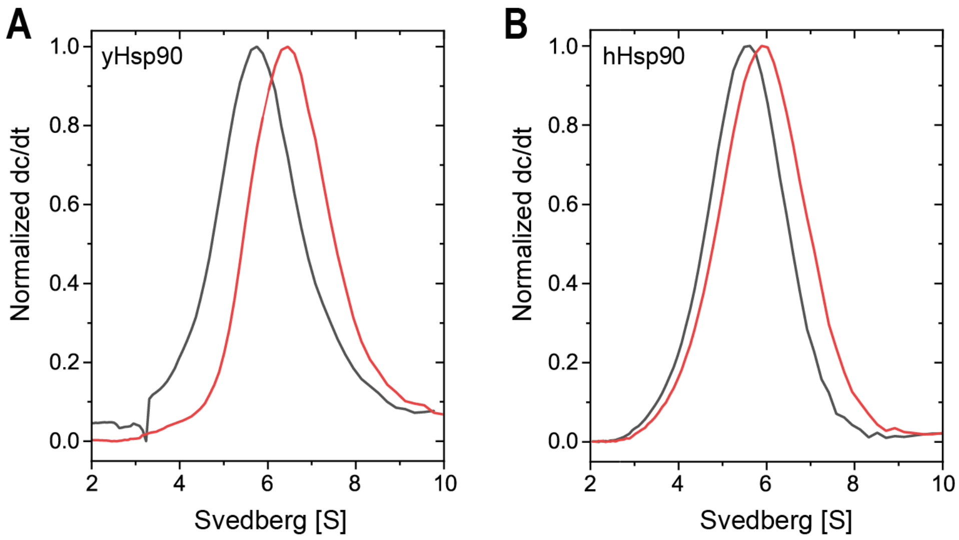

Supplementary Fig. 1: Closed state formation of labeled yHsp90 and hHsp90 determined by AUC.

A: Normalized *dc/dt* AUC sedimentation profiles of Atto550 labeled yHsp90 and (B) hHsp90. Proteins (1 µM) were incubated without nucleotide (black) or with 2 mM ATPγS (red) for 1 h in 200 mM KCl closing buffer (yHsp90 30°C; hHsp90 37°C).

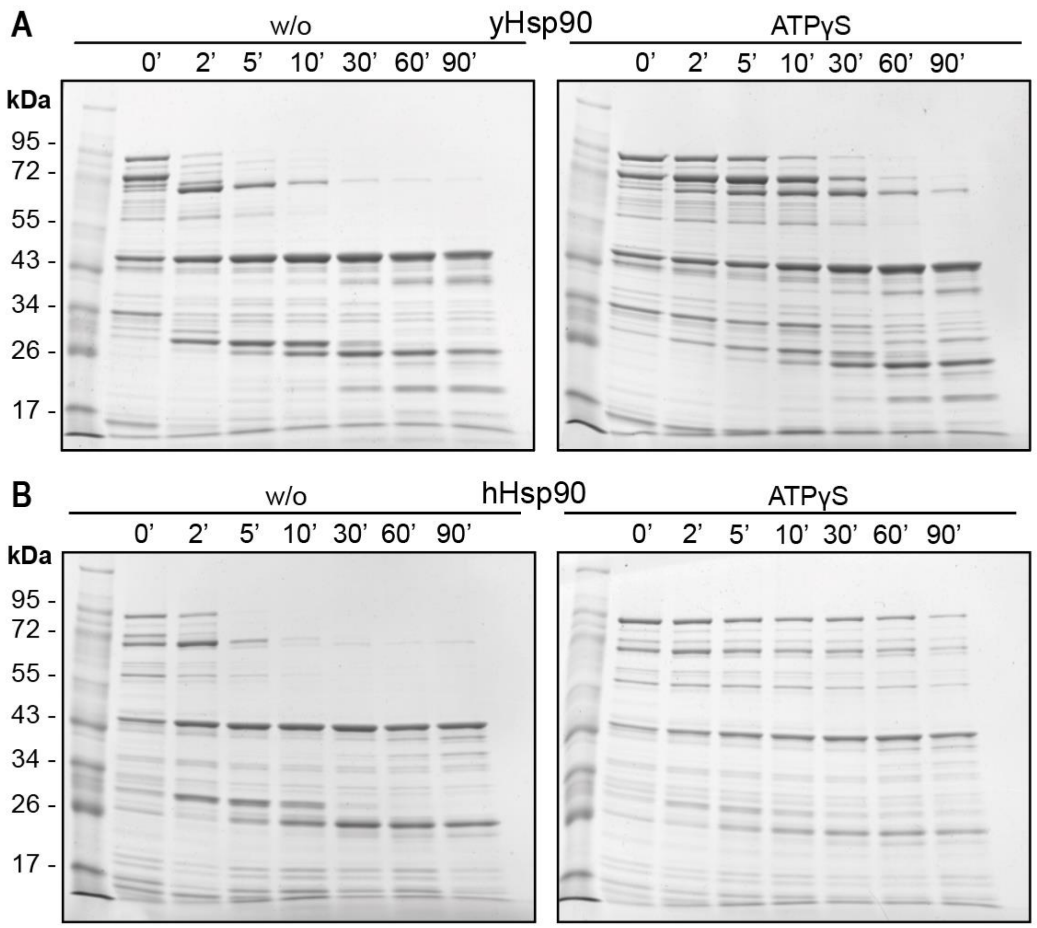

Supplementary Fig. 2: Conformational rearrangements of yHsp90 and hHsp90 characterized by limited proteolysis.

A: Yeast Hsp90 and (B) hHsp90 were subjected to the protease α-chymotrypsin (Hsp90:α-chymotrypsin 1:20). Proteolysis was conducted in 200 mM KCl closing buffer at room temperature. Proteins (0.3 mg/mL) were preincubated in the absence (left) or presence (right) of ATPγS (2 mM). The protease was uniformly added to every sample. The reaction was carried out at room temperature and stopped at several time points with 2 mM PMSF. Samples were mixed with 5x Laemmli buffer and analyzed by SDS-Page (4 – 12 % gradient).

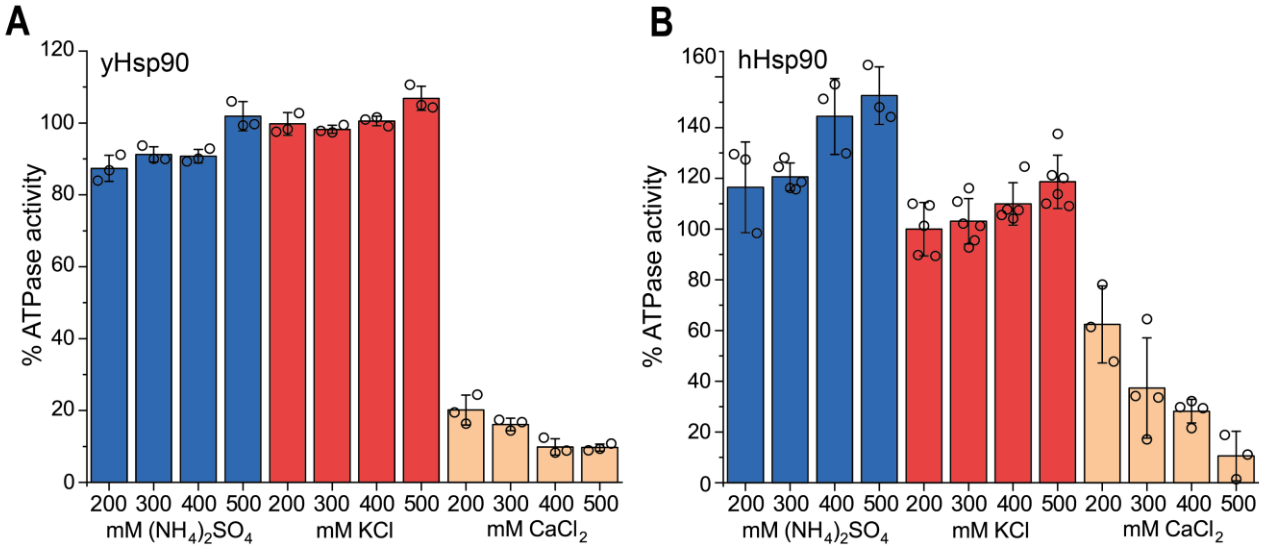

Supplementary Fig. 3: Comparison of yHsp90 and hHsp90 ATPase activities at varying buffer compositions.

ATPase activities of (A) yHsp90 and (B) hHsp90 measured *via* a regenerative ATPase assay. Activity was measured in closing buffer with different salts and concentrations (yHsp90: 30°C, 3 µM; hHsp90: 37°C, 10 µM). All measurements were performed at least as technical replicates (n =3) to calculate means and standard deviations. The obtained activity was corrected for background using Radicicol. Data was normalized to the obtained ATPase activity of the respective chaperone at 200 mM KCl.

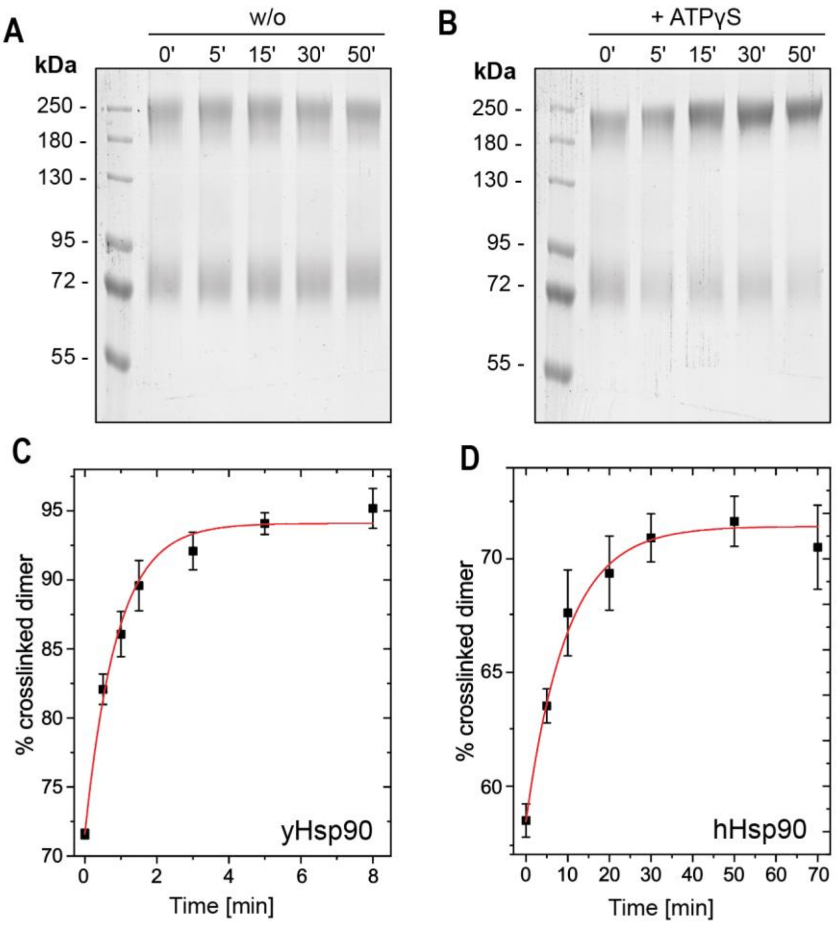

Supplementary Fig. 4: Closed-state formation kinetics of Hsp90 characterized by chemical crosslinking.

A: Exemplary SDS-PAGE analysis of crosslinked hHsp90 after different time points of incubation with ATPγS. Protein samples were incubated either in the absence or (B) in the presence of 2 mM ATPγS in closing buffer at 37°C. Chemical crosslinking was achieved by the addition of 2 mM DSG for 45 min. Subsequently, the reaction was quenched by the addition of 200 mM Tris. Samples were mixed with Laemmli buffer and analyzed by SDS-PAGE. C: Obtained closing kinetics of yHsp90 and (D) hHsp90 in 200 mM KCl closing buffer with 2 mM ATPγS. Measurements at various time points were carried out in triplicates (n = 3) to calculate means and standard deviations and fitted (yHsp90 $k_{closing}$ = 1.63 min^{-1}, hHsp90 $k_{closing}$ = 0.148 min^{-1}).

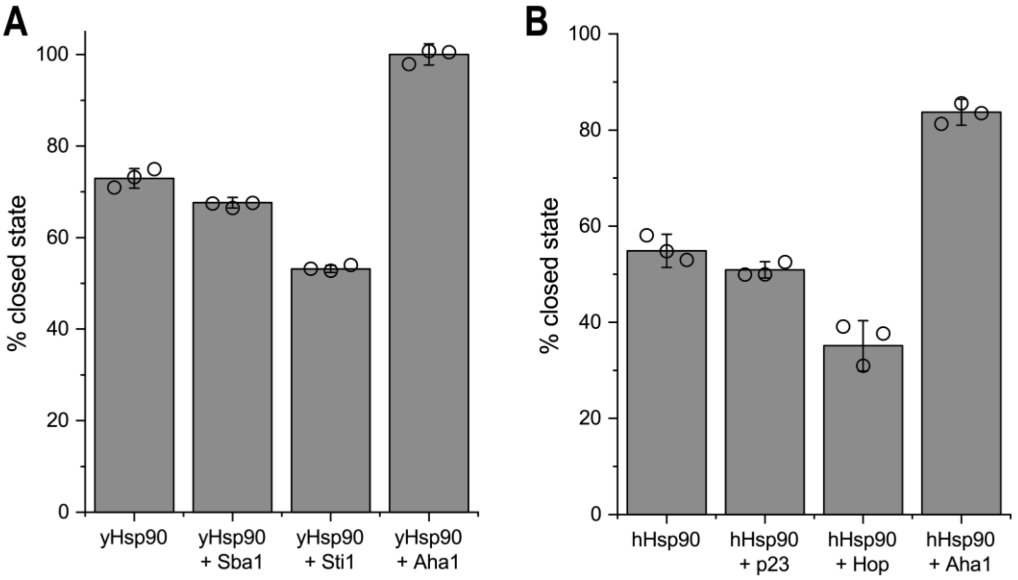

Supplementary Fig. 5: Influence of co-chaperones on the closing kinetics of Hsp90.

A: Yeast Hsp90 and (B) hHsp90 closed-state fraction after incubation with 2 mM ATPγS compared to protein additionally incubated with the respective co-chaperone (Hsp90:Co-chaperone = 1:2). Incubation was carried out for 1 min at 30°C for yHsp90 and for 10 min at 37°C for hHsp90. The data shown was obtained by fitting the SEC elution profiles with a bi-Gaussian fit. All measurements were performed as technical replicates (n = 3) to calculate means and standard deviations.

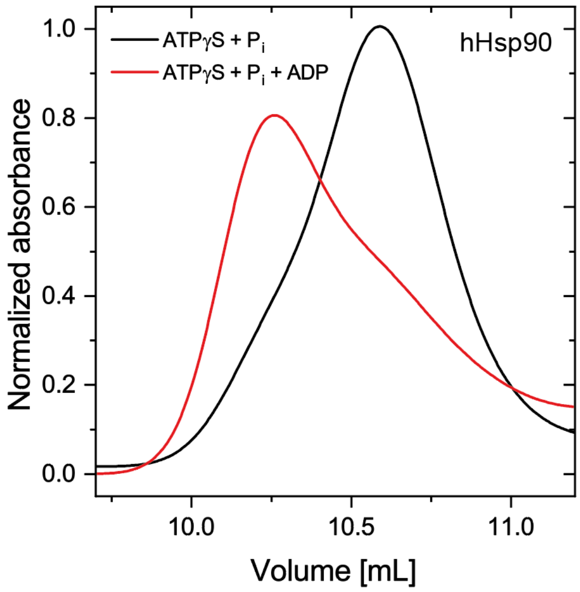

Supplementary Fig. 6: Impact of inorganic phosphate on closing and re-opening.

Normalized hHsp90 elution profile from SEC after incubation in 200 mM KCl closing buffer 37°C, 1 h) with 2 mM ATPγS and in the presence of 5 mM phosphate to initiate the closed state (black). Afterwards, ADP (red) was added and incubated for 1 h at 37°C to probe dimer re-opening.

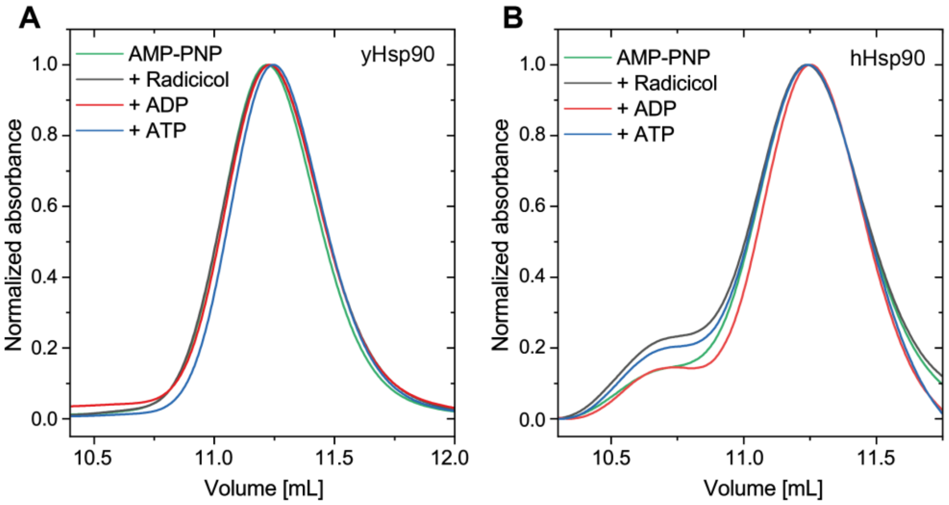

Supplementary Fig. 7: Re-opening of AMP-PNP closed yHsp90 and hHsp90 characterized by SEC.

A: Normalized yHsp90 and (B) hHsp90 elution profiles from SEC after incubation in 200 mM KCl closing buffer (yHsp90: 30°C, 1 h; Hsp90: 37°C, 16 h) with 2 mM AMP-PNP (green) to initiate the closed state. Afterwards, ATP (blue), ADP (red) or Radicicol (black) were added and incubated for 1 h to probe dimer re-opening.

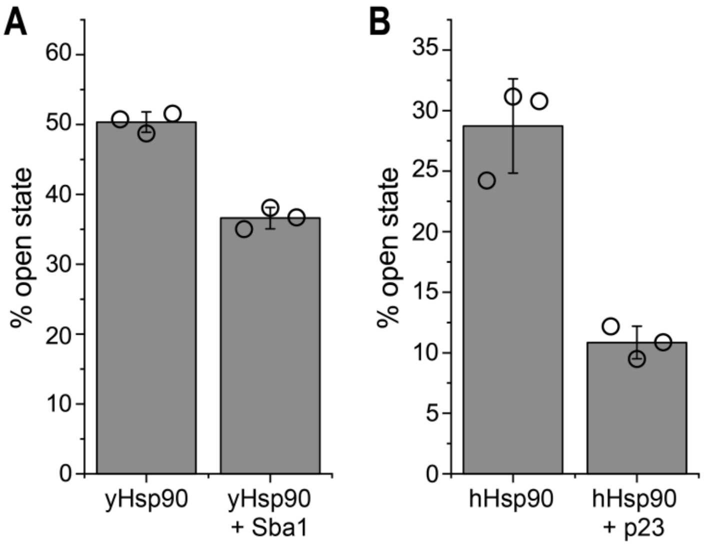

Supplementary Fig. 8: Influence of the co-chaperone Sba1/p23 on dimer re-opening of Hsp90.

A: yHsp90 open-state fraction after 10 min of incubation with radicicol (500 µM) either in the absence or in the presence of Sba1 (yHsp90:Sba1 = 1:2). The protein was incubated before in 200 mM KCl closing buffer (1 h at 30°C) with 2 mM ATPγS to initiate the closed state. B: hHsp90 re-opening with radicicol (500 µM) compared to protein incubated with p23 after 10 min of incubation at 37°C. The protein was incubated before in 200 mM KCl closing buffer (1h at 37°C) with 2 mM ATPγS to initiate the closed state. The data shown was obtained by fitting the SEC elution profiles with a bi-Gaussian fit. All measurements were performed as independent technical replicates (n = 3) to calculate means and standard deviations

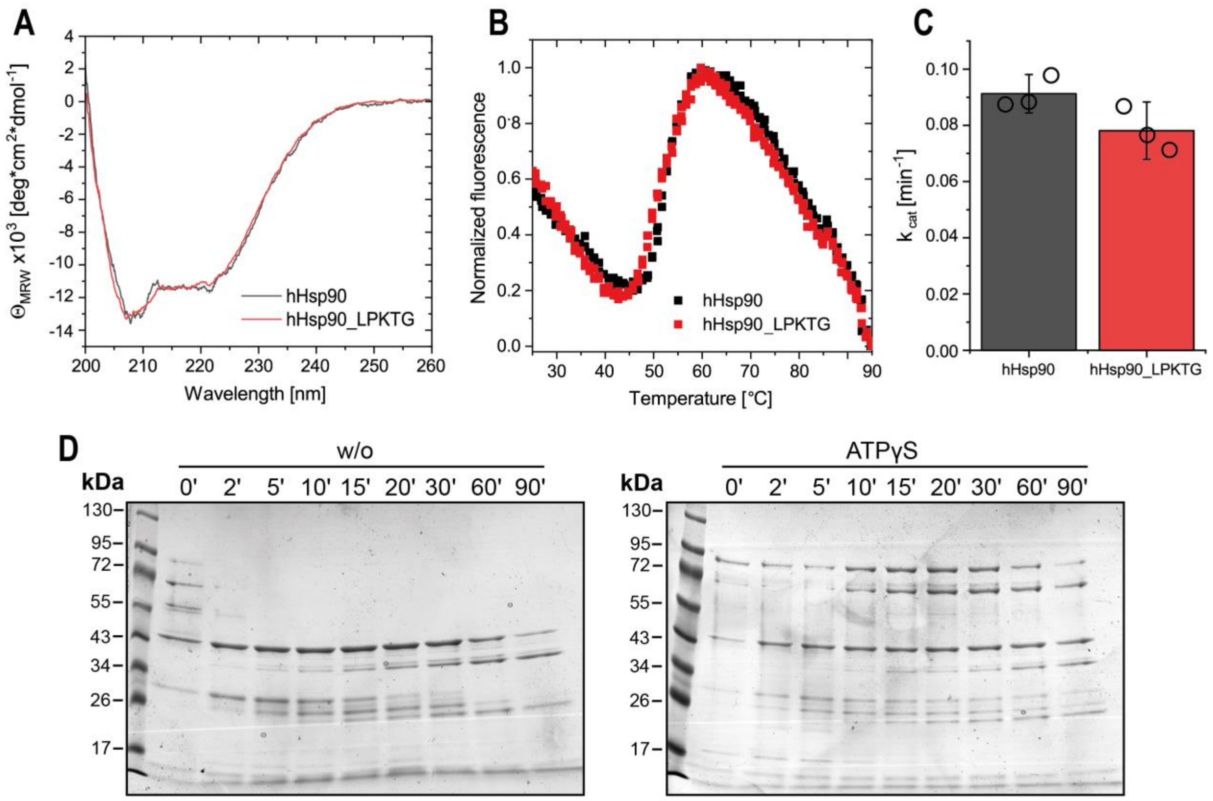

Supplementary Fig. 9: *In vitro* characterization of hHsp90_LPKTG and comparison to wt hHsp90.

A: The secondary structure of wt hHsp90 was compared to hHsp90_LPKTG by far UV spectroscopy. Spectra were recorded at a concentration of 0.2 mg/mL in 50 mM phosphate buffer (pH 7.4) at room temperature. B: Thermal stability of the proteins was compared by a thermal shift assay with Sypro orange. The assay was conducted in HKM buffer with 0.25 mg/mL of protein at a heating rate of 1°C/min. C: Comparison of ATPase activity of wt and mutated Hsp90. Hydrolysis rate was measured using a regenerative ATP system. The assay was performed in HKM buffer (40 mM HEPES, pH 7.4, 150 mM KCl, 5 mM $MgCl_2$) with 2 mM ATP and 10 µM of Hsp90 at 37°C. Measurements were performed as independent technical replicates (n = 3) to calculate means and standard deviations. The obtained activity was corrected for background using Radicicol. D: Mutated Hsp90, containing the LPKTG motif in the charged linker, was subjected to the protease α-chymotrypsin (Hsp90:α-chymotrypsin 1:20). Proteolysis was conducted in HKM buffer at room temperature in the absence (left) or presence (right) of ATPγS. The reaction was stopped at the time points indicated with 2 mM PMSF and analyzed by SDS-Page analysis (4 – 12 % gradient).

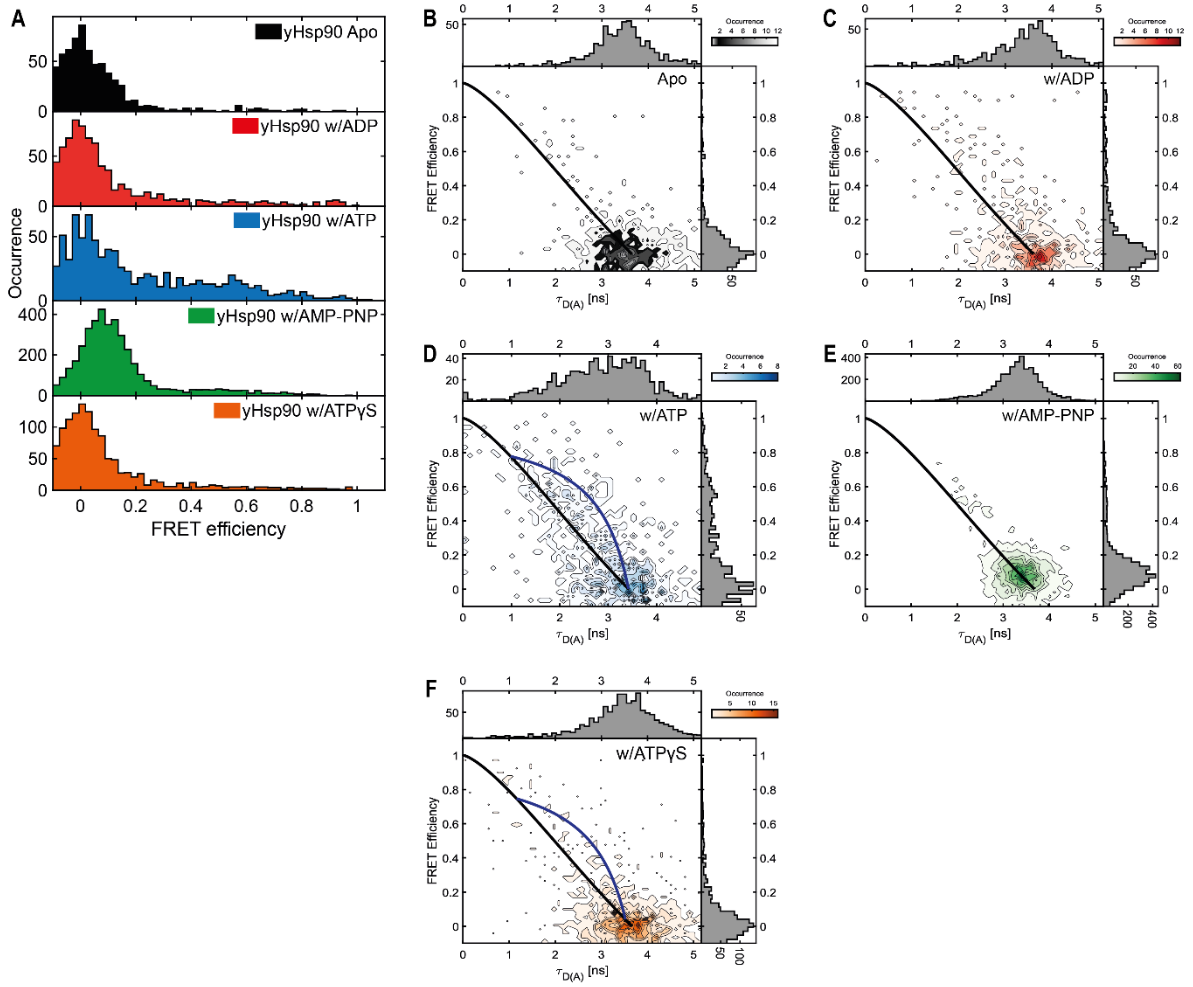

Supplementary Fig. 10: Overview of yHsp90 smFRET measurements and lifetime analysis

A: FRET efficiency histograms of yHsp90 in the apo state (black) and in the presence of ADP (red), ATP (blue), AMP-PNP (green) and ATPγS (orange). FRET histograms are representatives from at least two independent measurements unless otherwise mentioned. B-F: 2D FRET efficiency vs. donor fluorescence lifetime in the presence of acceptor ($\tau_{D(A)}$) plots of B: apo yHsp90, C: w/ADP, D: w/ATP, E: w/AMP-PNP and F: w/ATPγS. The black lines indicate the static FRET line, whereas blue curved lines depict the dynamic FRET line. Nucleotides were preincubated for 2 hours and concentrations were kept at 2 mM for all measurements. The values from the fits to the fluorescence lifetime distributions used to determine the dynamic FRET lines are given in Table S1.

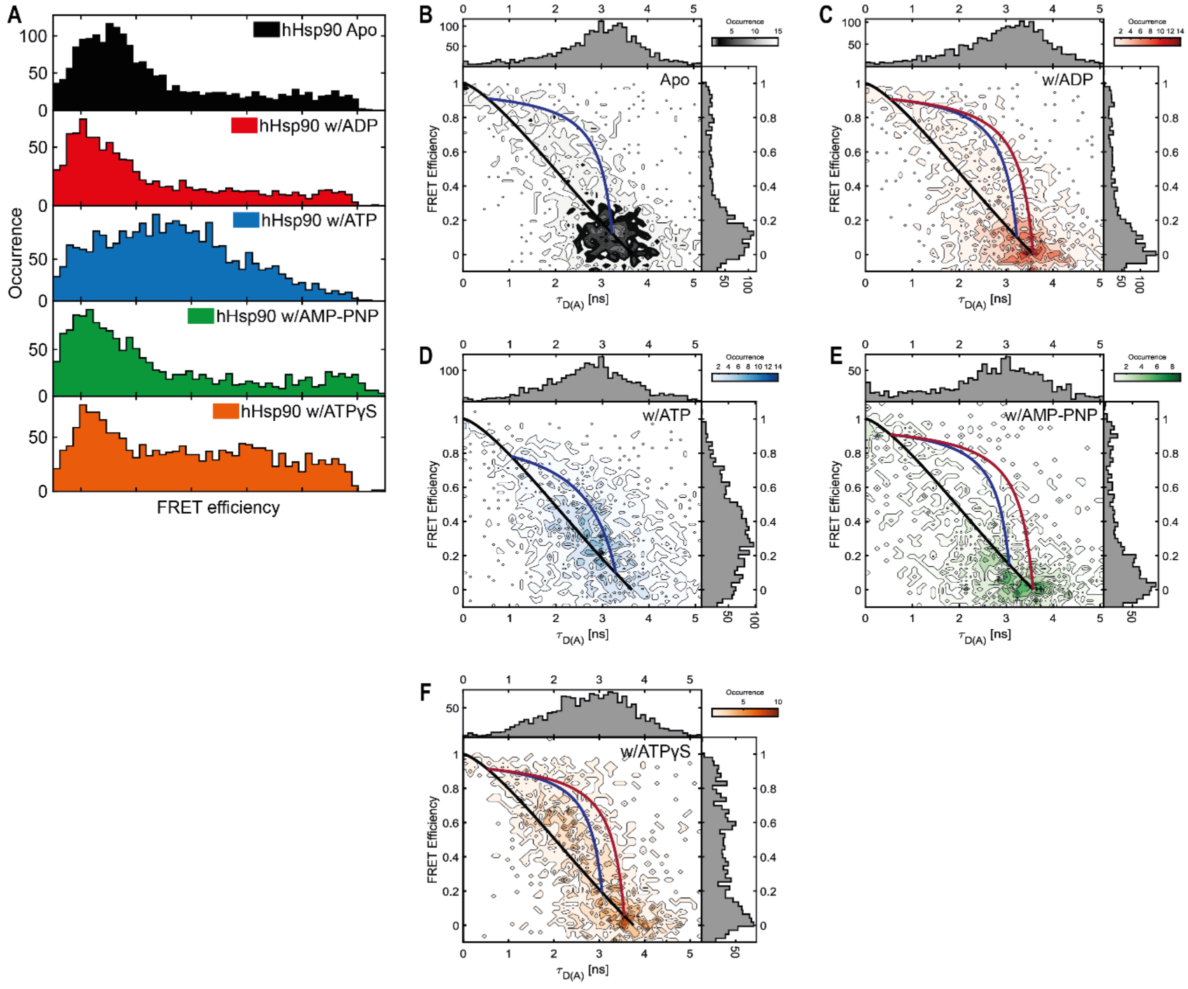

Supplementary Fig. 11: Overview of the hHsp90 smFRET measurements and lifetime analysis

A: FRET efficiency histograms of hHsp90 in the apo state (black), in the presence of ADP (red), ATP (blue), AMP-PNP (green) and ATPγS (orange). FRET histograms are of representatives from at least two independent measurements unless otherwise mentioned. B-F: 2D FRET efficiency vs. donor fluorescence lifetime in the presence of acceptor ($\tau_{D(A)}$) plots of B: *apo* hHsp90, C: w/ADP, D: w/ATP, E: w/AMP-PNP and F: w/ATPγS. The black lines indicate the static FRET line, whereas blue curved lines depict the dynamic FRET line. Nucleotides were preincubated for 4 hours and concentrations were kept at 2 mM for all the measurements. The values from the fits to the fluorescence lifetime distributions used to determine the dynamic FRET lines are given in Supplementary Table 1.

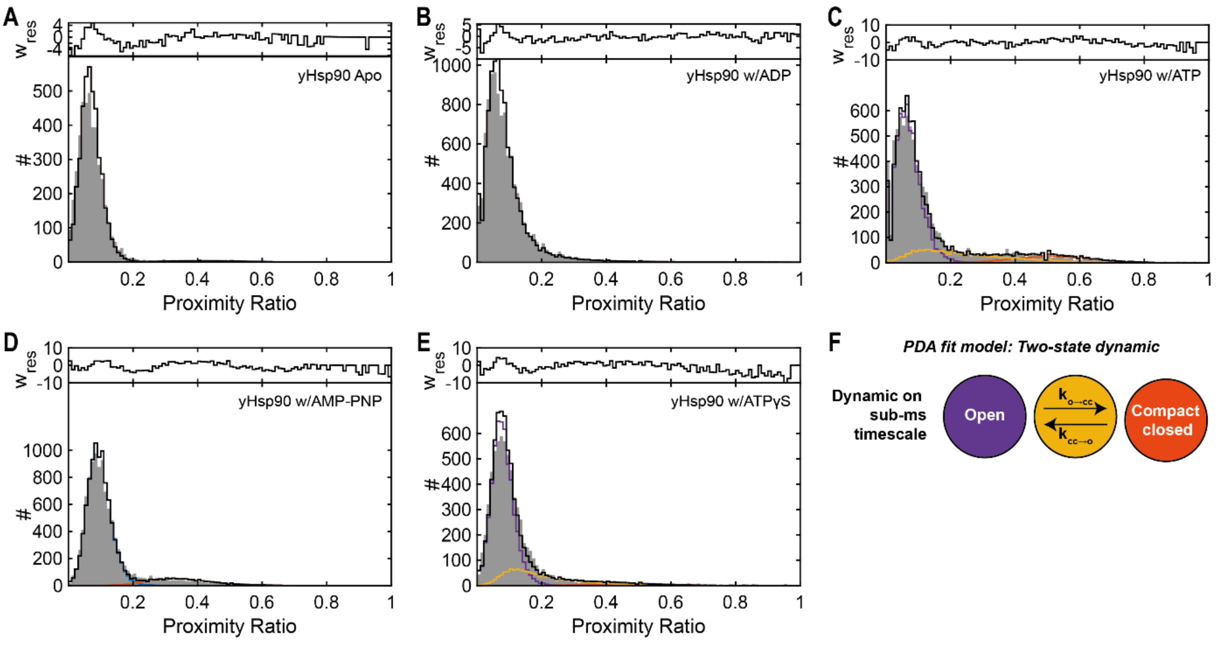

Supplementary Fig. 12: Dynamic photon distribution analysis (PDA) of yHsp90 smFRET measurements.

For dynamic PDA, uncorrected FRET efficiency (proximity ratio) histograms with time binnings of 0.5. 1.0, 1.5 and 2.0 ms were used to calculate the distance distribution for different FRET populations and the dynamics between them. Histograms with 1 ms binning are shown here for yHsp90 in the *apo* state (A), and in the presence of ADP (B), ATP (C), AMP-PNP (D) and ATPγS (E). All nucleotides were incubated at a concentration of 2 mM for 2 h before the smFRET experiments. F: Schematic representation of the two-state dynamic PDA fit model used for yHsp90 w/ATP and w/ATPγS conditions where dynamic interconversion between open (purple) and closed-compact (red) states were present. The contribution of the dynamically interconverting population is shown in yellow. A two-state static model was used for yHsp90 apo and w/AMP-PNP conditions while a single state was detected in the presence of ADP. Values are given in Supplementary Table 2.

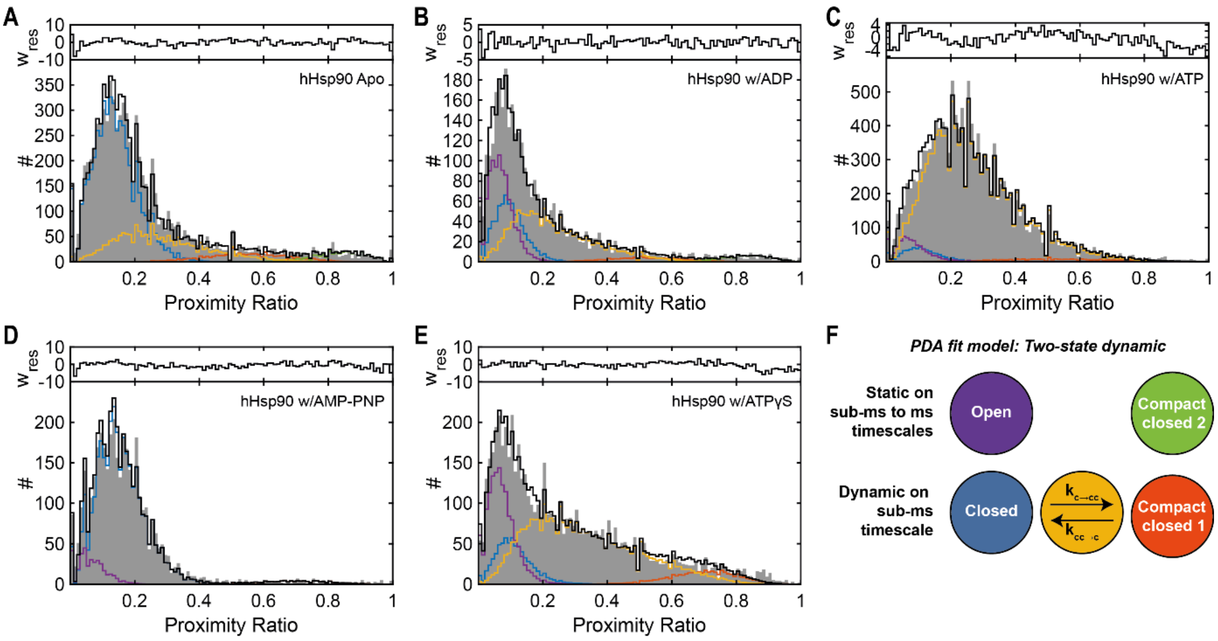

Supplementary Fig. 13: Dynamic photon distribution analysis (PDA) of hHsp90 smFRET measurements.

For dynamic PDA, uncorrected FRET efficiency (proximity ratio) histograms with time binnings of 0.5. 1.0, 1.5 and 2.0 ms were used to calculate the distance distribution for different FRET populations and the dynamics between them. Histograms with 1 ms binning are shown here for hHsp90 in the apo state (A), and in the presence of ADP (B), ATP (C), AMP-PNP (D) and ATPγS (E). All nucleotides were incubated at a concentration of 2 mM for 4 h before the smFRET experiments. F: Schematic representation of the two-state dynamic PDA fit model used for hHsp90 in the apo state and in the presence of ADP, ATP and ATPγS where dynamic interconversion between a closed (blue) and a closed-compact 1 (red) state was present. The contribution of the dynamically interconverting population is shown in yellow. The presence of the open state (violet) was also included in the fits except for the hHsp90 apo. An additional static high FRET state (closed compact 2, green) was included in the two-state dynamic PDA fits for hHsp90 apo and hHsp90 in the presence of ADP. For hHsp90 in the presence of AMP-PNP, a three-state static model was used for the PDA analysis where open, closed and closed-compact 1 states were present. Values are given in Supplementary Table 2.

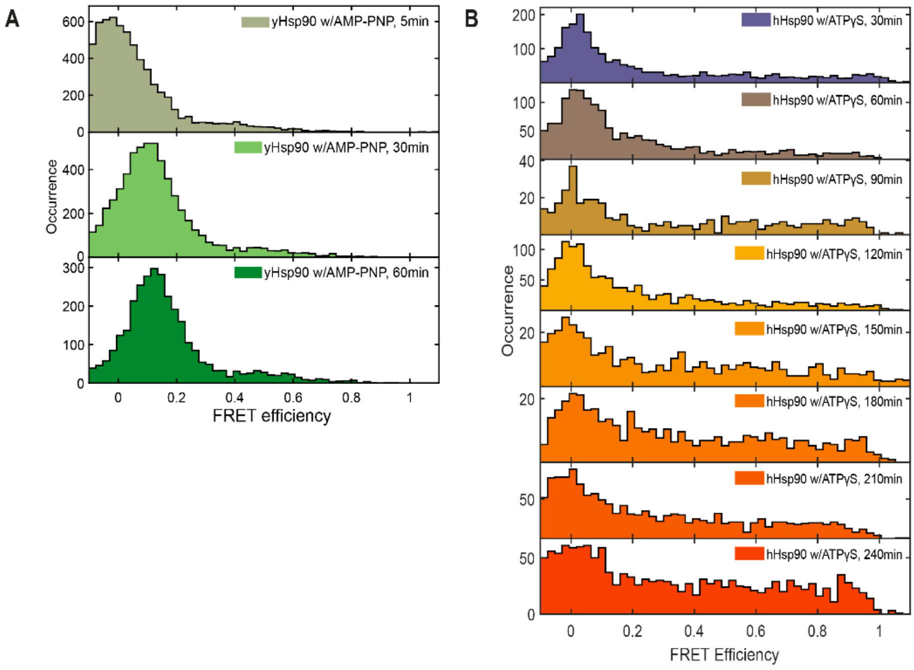

Supplementary Fig. 14: Kinetic measurements for both Hsp90s.

A: The closing kinetics of yHsp90 in the presence of AMP-PNP assessed by smFRET efficiency histograms after 5, 30 and 60 minutes of incubation with the nucleotide. B: SmFRET efficiency histograms for the closing kinetics of hHsp90 in the presence of ATPγS. SmFRET measurements were performed after 30, 60, 90, 120, 150, 180, 210, and 240 min from the start of the incubation with ATPγS. FRET histograms are of representatives from at least two independent measurements unless otherwise mentioned.

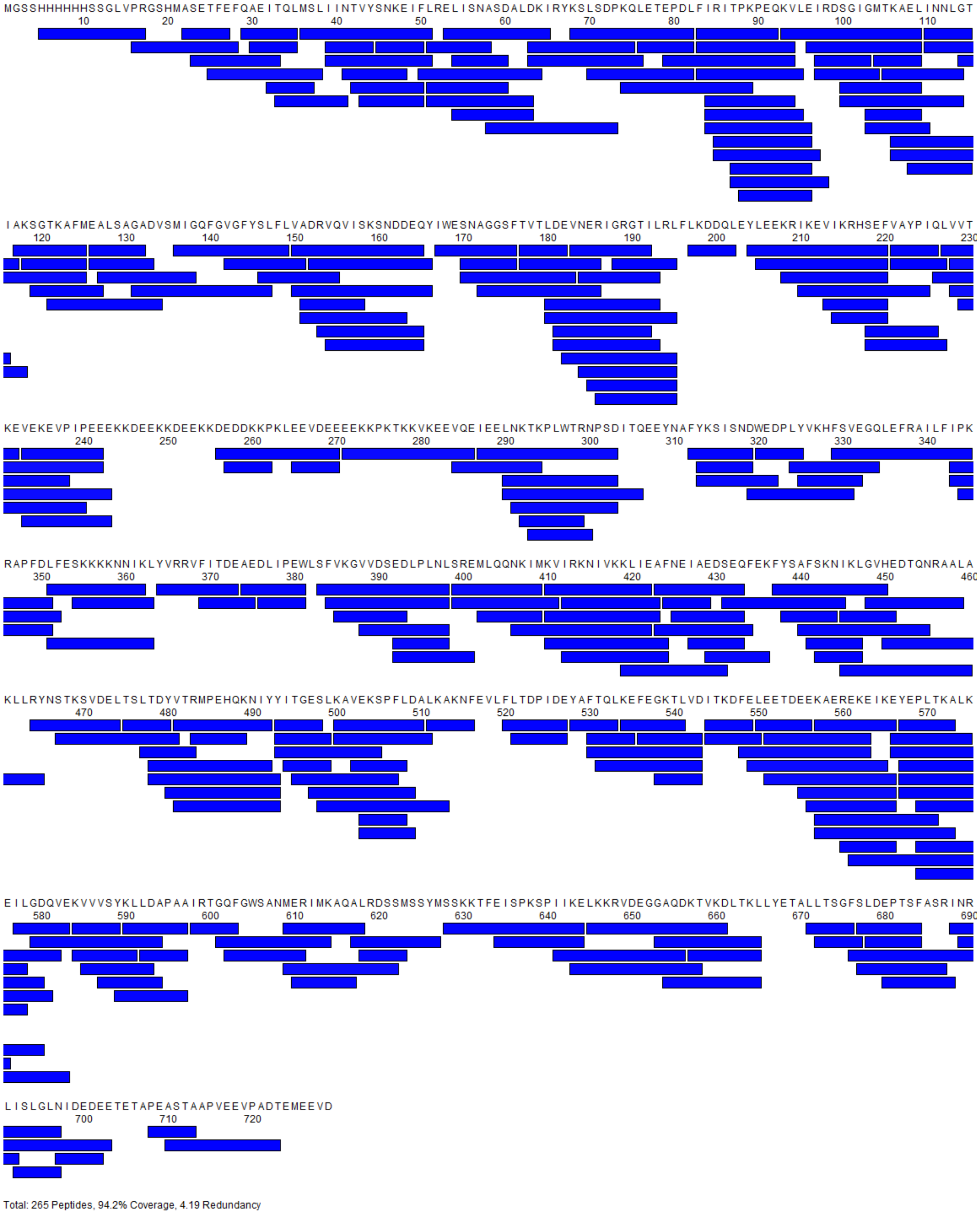

Supplementary Fig. 15: HDX peptide coverage of yHsp90.

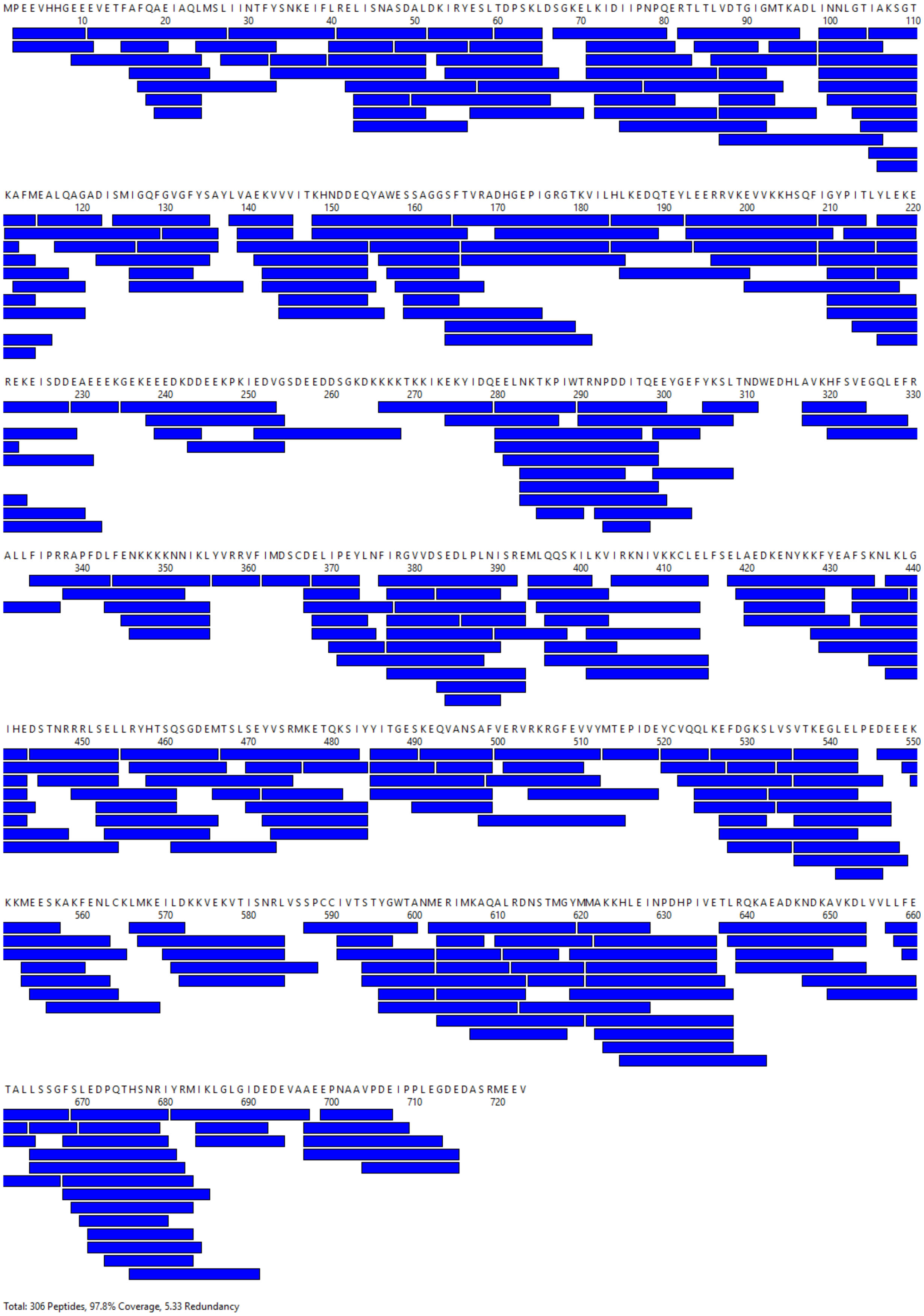

Supplementary Fig. 16: HDX peptide coverage of hHsp90.

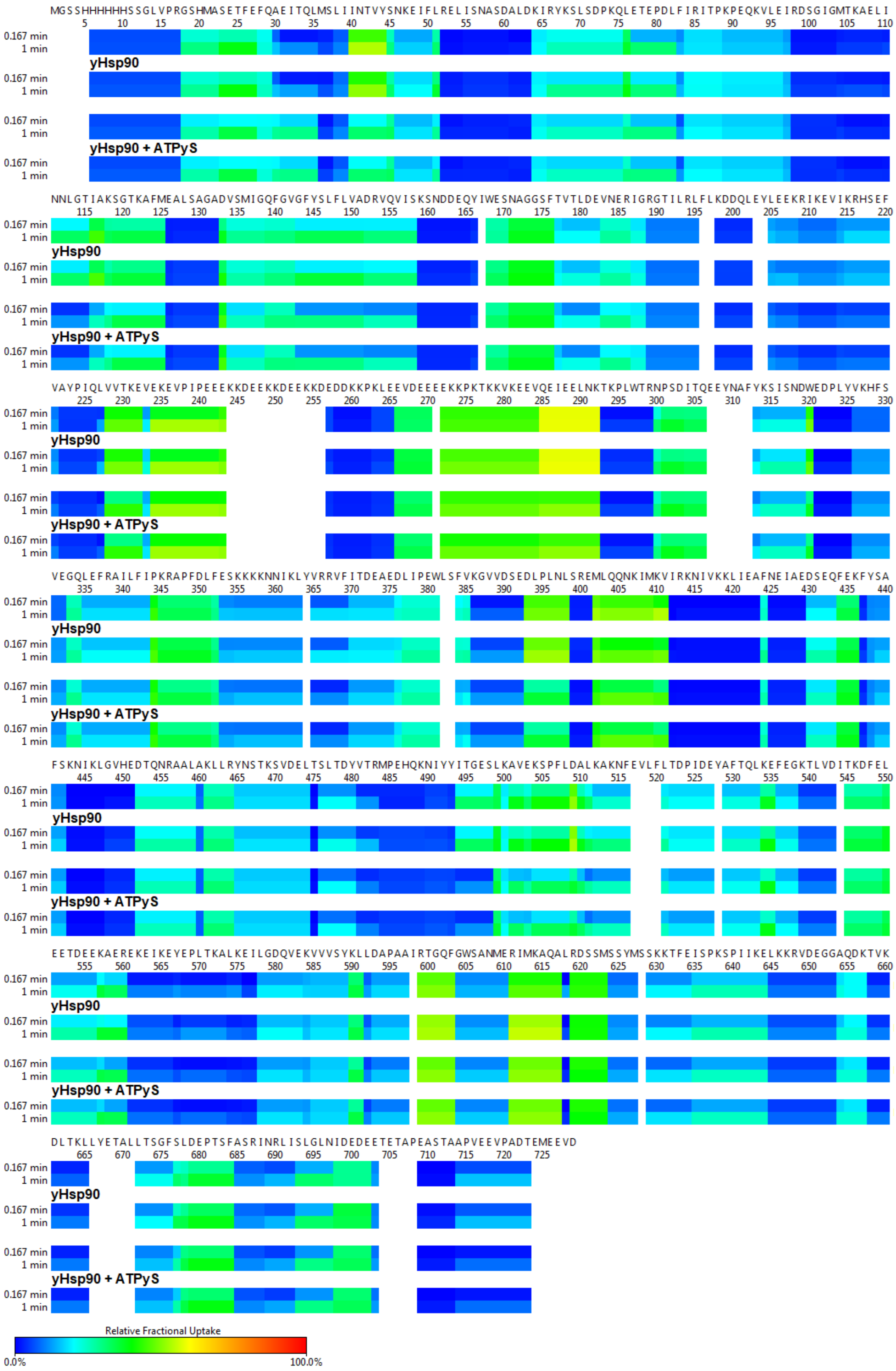

Supplementary Fig. 17: HDX relative fractional uptake by residue (yHsp90).

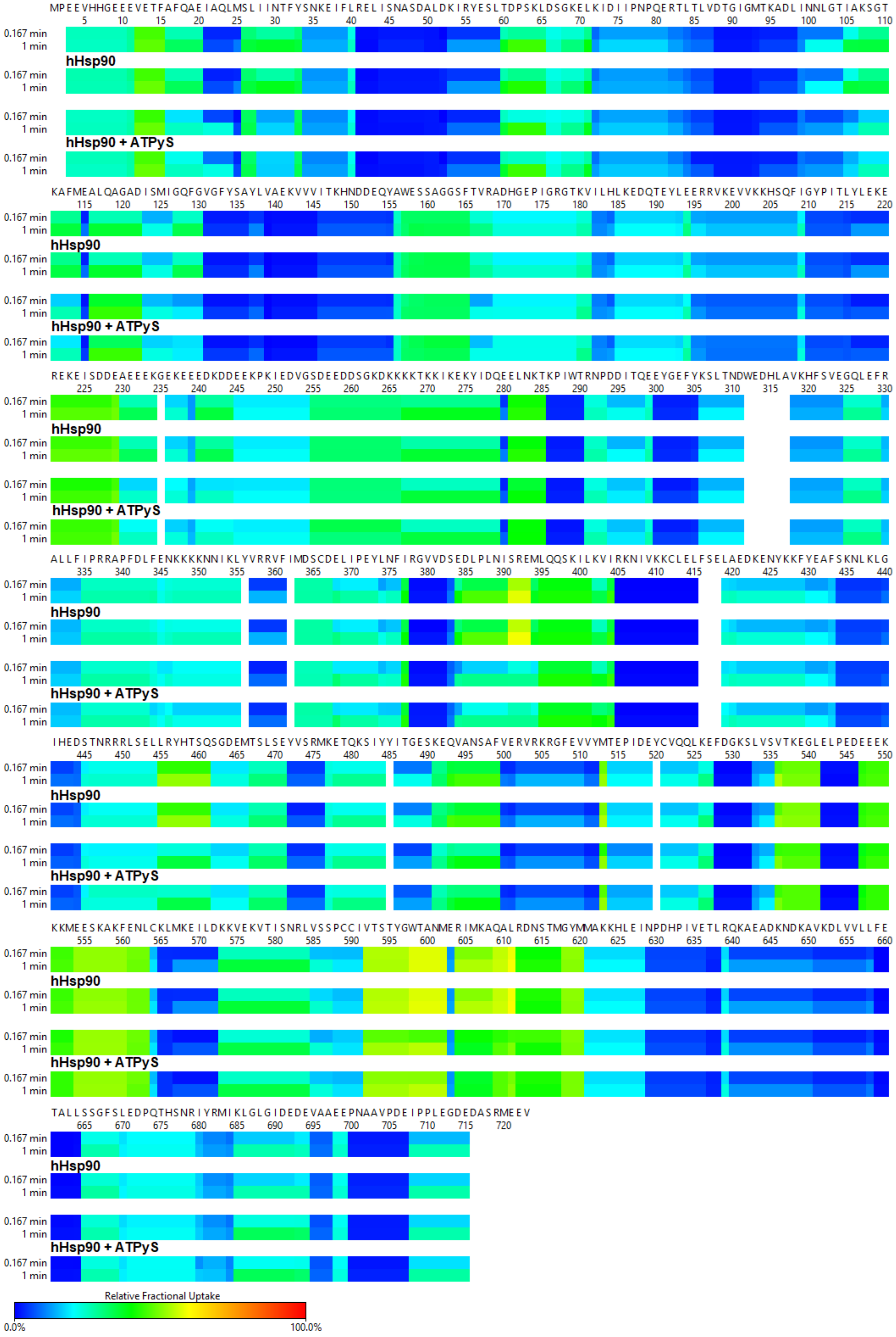

Supplementary Fig. 18: HDX relative fractional uptake by residue (hHsp90).

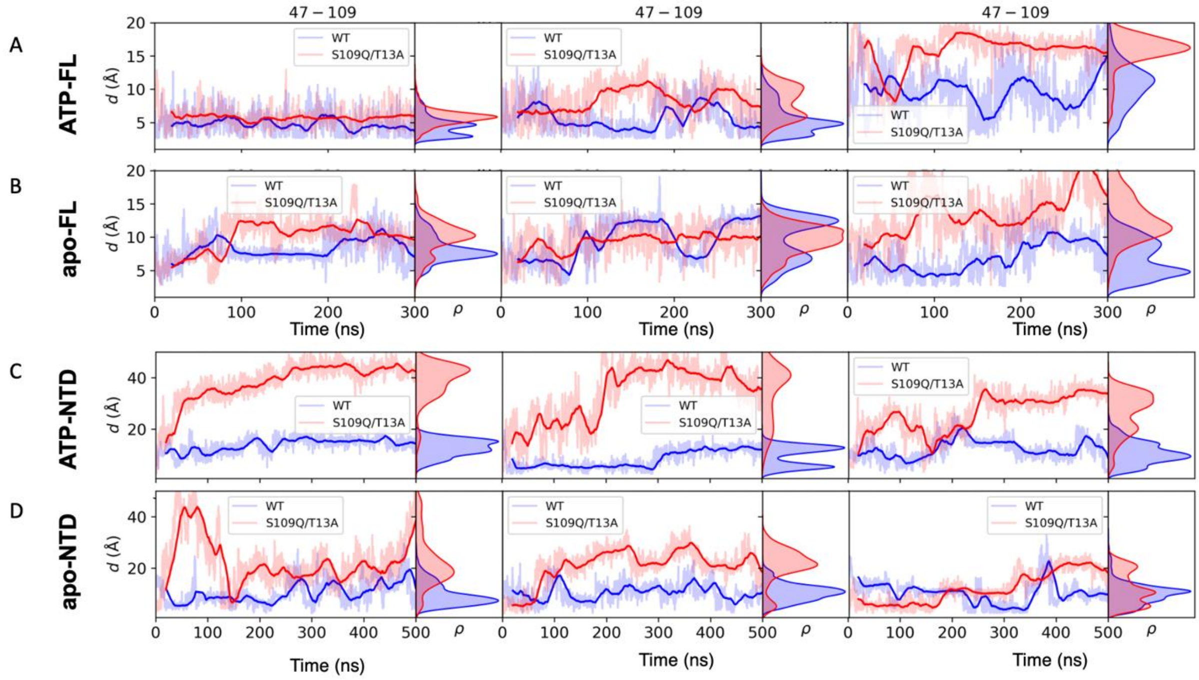

Supplementary Fig. 19: Analysis of MD simulations of the WT and S109Q/S13A mutant of the full-length (FL) and NTD of yHsp90.

The figure shows independent replicas of the ATP lid dynamics measured by the Tyr47-Ser/Gln109 distance in Å for; A: ATP-bound state of FL-yHsp90, B: FL *apo* state of FL-yHsp90, C: ATP-bound state of NTD-yHsp90, and D: FL *apo* state of NTD-yHsp90.

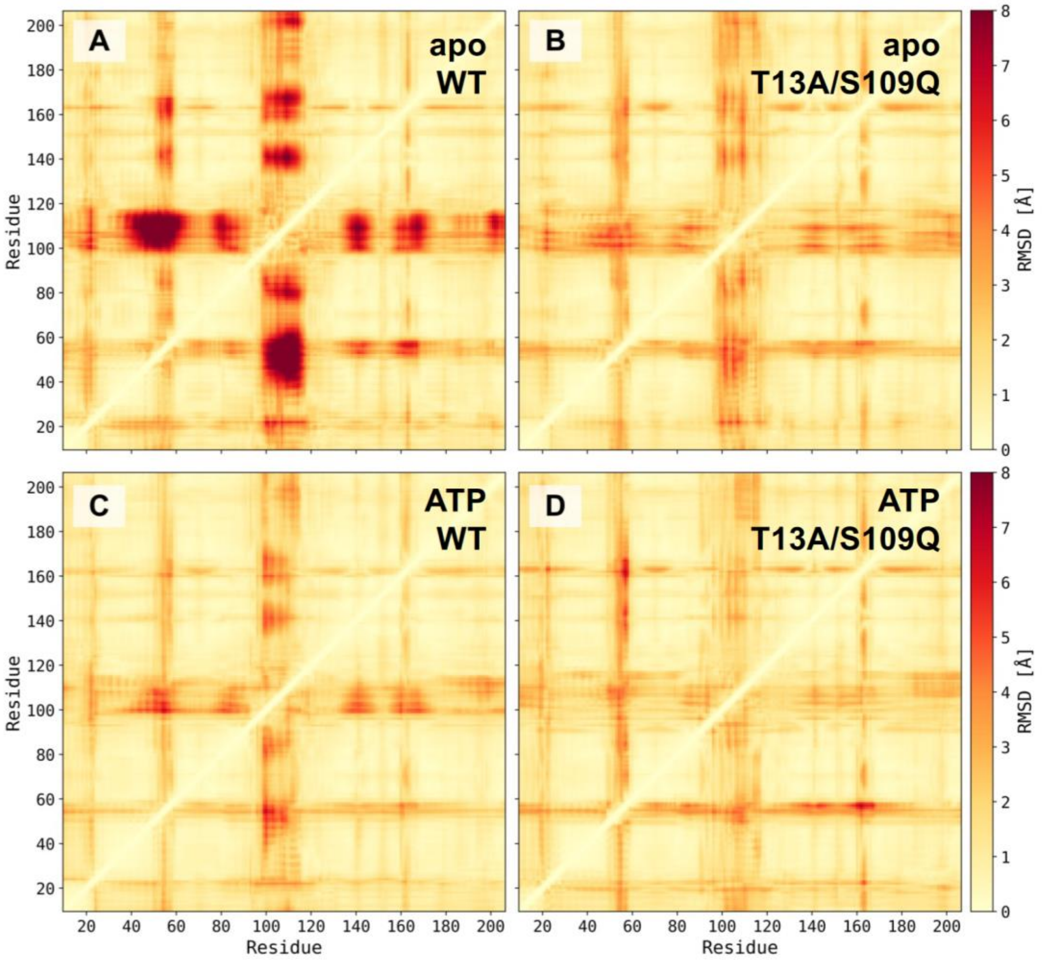

Supplementary Fig. 20: Conformational dynamics of Hsp90 explored by MD simulations.

Root-mean-square-deviation (RMSD) of Cα-Cα pair distances for the NTD region obtained from MD simulations of full-length yHsp90 (A: *apo* and C: ATP-bound state) and T13A-S109Q mutant (B: *apo* and D: ATP-bound state). Darker colours correspond to regions with higher conformational flexibility. Changes in the conformational flexibility upon ATP binding could contribute to differences between the yeast and human Hsp90.

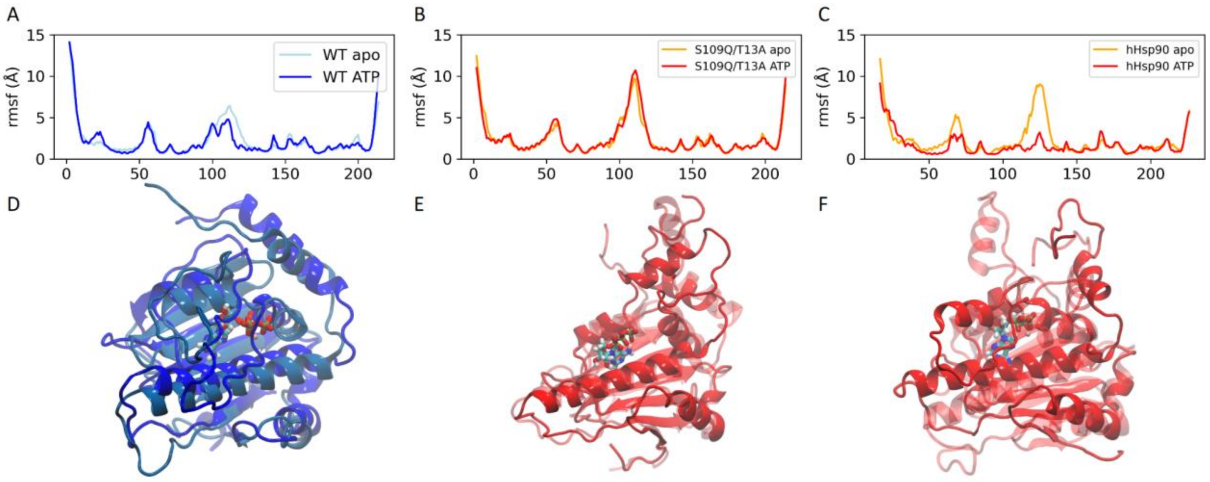

Supplementary Fig. 21: MD simulations of the NTD.

A-C: Root-mean-square-fluctuations (rmsf) of the NTD indicate that the *apo* states are generally more flexible than the ATP-bound form. The rmsf per residue are shown for; A: yHsp90 in the *apo* (light blue) and ATP-bound (blue) states. B: yHsp90-S109Q/S13A in the *apo* (orange) and ATP-bound (red) states. The mutation induces the opening of the NTD. C: The rmsf per residue are shown for hHsp90 in the *apo* (orange) and ATP-bound (red) states. D-F: Snapshots from MD simulations of the ATP-bound (full colour) and the *apo* state (transparent colour) for D: WT-yHsp90, E: S109Q/S13A-yHsp90, and F: hHsp90.

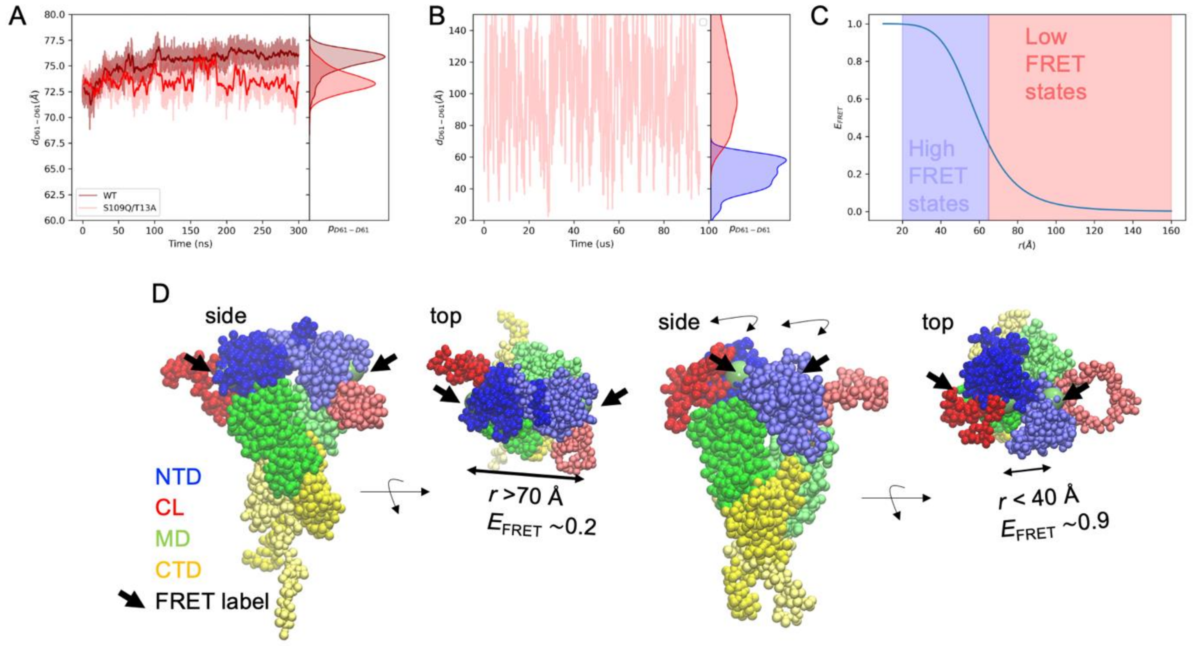

Supplementary Fig. 22: Comparison of molecular simulations with smFRET experiments.

A: The D61-D61 distances, used as a proxy for the measured FRET efficiencies, taken from the atomistic molecular dynamics simulations suggest that the WT and T13A/S109Q of the FL-yHsp90 remain in an overall similar conformation to the closed-state X-ray structure (PDB ID: 2CG9). The distances are comparable to a state with ca. 8% FRET efficiency (see panel C), when including the additional contribution of the linkers and fluorophores. The T13A/S109Q mutation introduces a subtle local increase in the E_{FRET} relative to the WT-yHsp90.

B: Coarse-grained molecular dynamics (cgMD) simulations on ~100 μs timescales show local dissociation and rotation of the NTDs leading to states with a short D61-D61 distance (< 65 Å), comparable with a high efficiency FRET (>40%).

C: General dependence of the FRET efficiency as a function of donor-acceptor separation with R_0=59 Å, based on $E_{\mathrm{FRET}} = 1/[1+(r/R_0)^6]$.

D: Snapshots from the cgMD simulations showing the canonical closed form of Hsp90 (*left*, comparable to E_{FRET}~0.2), and a structure with rotated NTDs (right, comparable to E_{FRET}~0.9). See Refs. [1-3] for further discussion on the NTD dissociation and rotation.

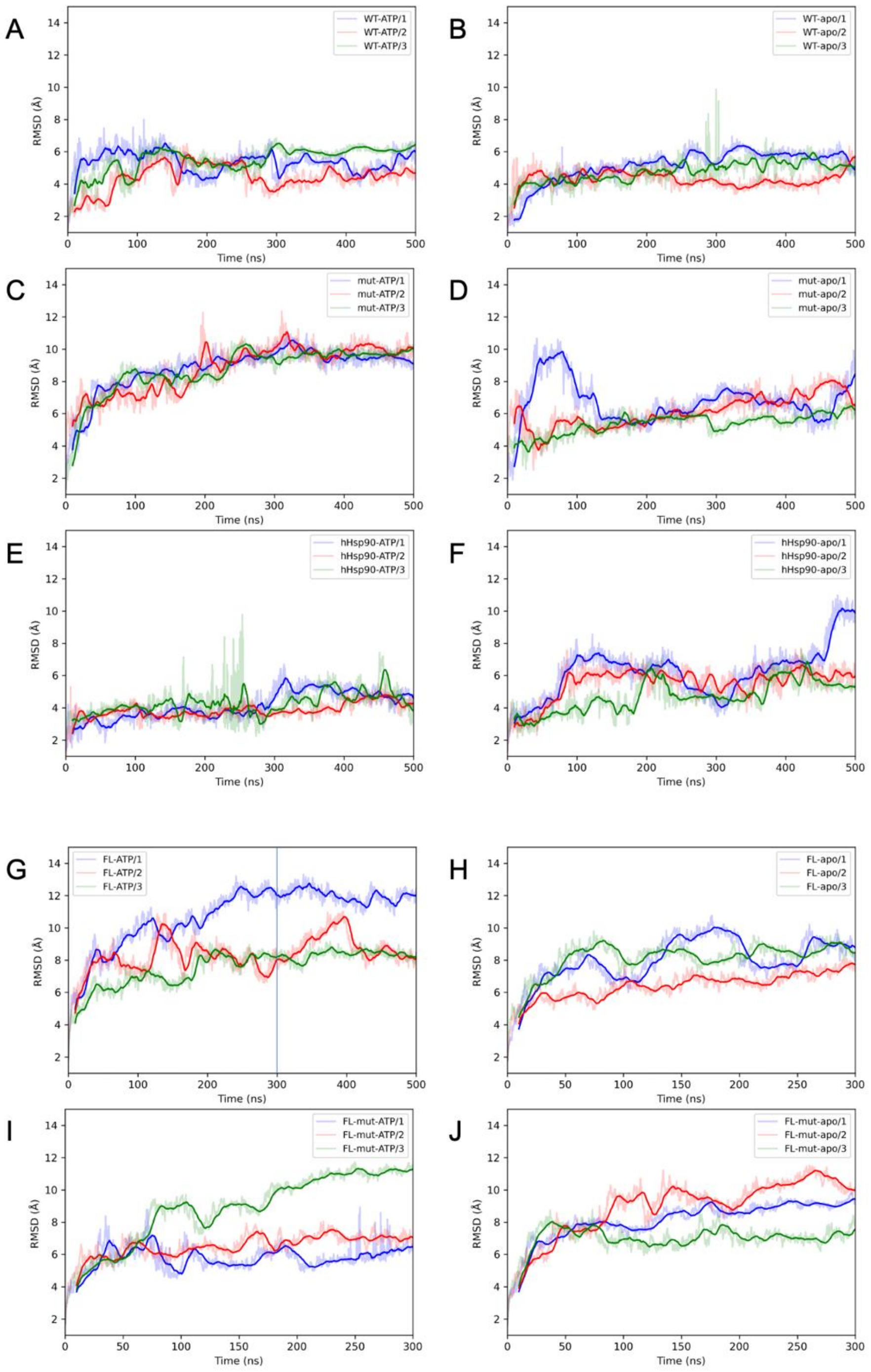

Supplementary Fig. 23: Root-mean-square-deviations (RMSD) during molecular dynamics simulations.

The figure shows that the RMSD of the protein backbone locally stabilizes during the first few hundred nanoseconds of the simulation. A, B: RMSD of the NTD of WT-yHsp90 in (A) ATP and (B) *apo* states. C, D: RMSD of the NTD of T13A/S109Q-yHsp90 in (C) ATP and (D) *apo* states. E, F: RMSD of the NTD of WT-hHsp90α in (E) ATP and (F) *apo* states. G, H: RMSD of the FL WT-yHsp90 in (G) ATP and (H) *apo* states. Extension of the simulations from 300 ns to 500 ns leads to small fluctuations in the overall structure, supporting that the (local) dynamics has converged. I, J: RMSD of the FL T13A/S109Q-yHsp90 in (I) ATP and (J) *apo* states.

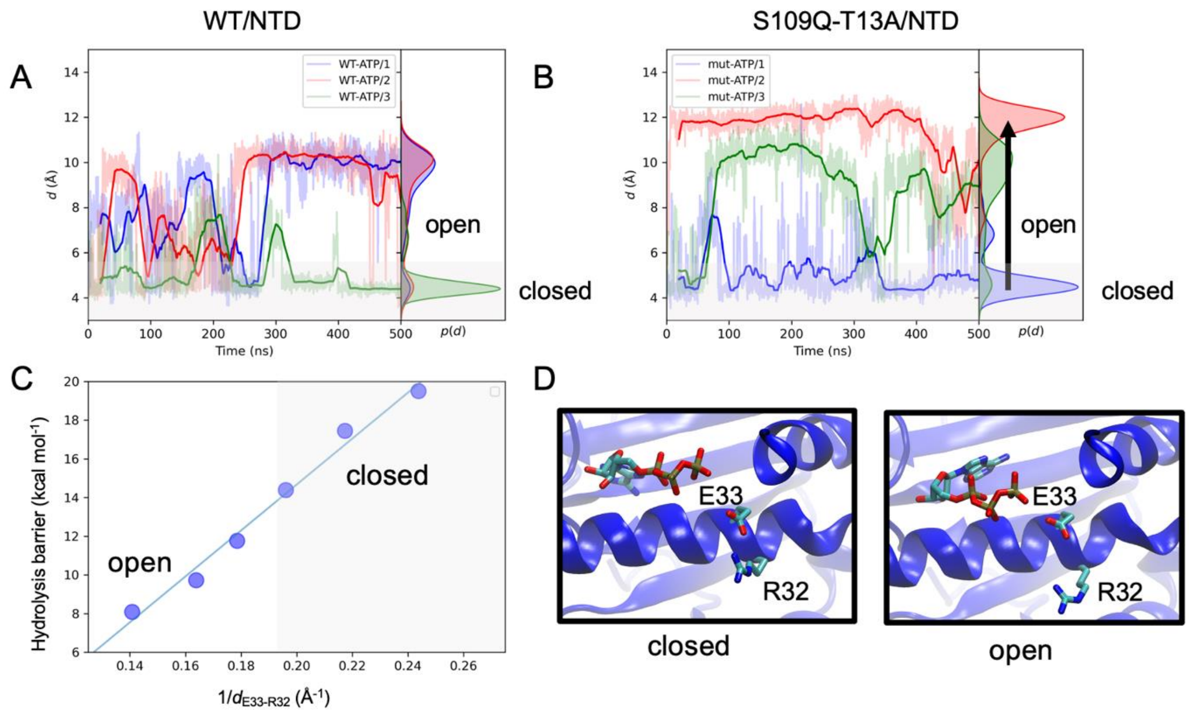

Supplementary Fig. 24. Dynamics of catalytically important elements in Hsp90.

A, B: Dynamics of the R32-E33 ion-pair in A) WT-NTD with ATP and B) S109Q-T13A-NTD with ATP. The S109Q-T13A substitution affects the dynamics of the R32-E33 ion-pair, leading to an increased population of the open ion-pair conformation.

C: Dependence of barrier for ATP hydrolysis on the R32-E33 distance from density functional theory (DFT) calculations (B3LYP-D3/def2-TZVP level). Data based on Ref. [4].

D: Snapshots on closed and open ion pair conformations from molecular dynamics simulations.

Yeast Hsp90	τ_1 ***(ns)***	f_{c1}	τ_2 ***(ns)***	f_{c2}	***Open conformation***	f_o	χ^2_{red}
**Apo**					3.43	1	1.72
**ADP**					3.61	1	1.57
**ATP**	0.98	0.09			3.49	0.91	1.65
**AMP-PNP**			3.41	1	3.68		1.72
**ATPγS**	1.16	0.12	3.52	0.88	3.67	0.88	1.64
Human Hsp90							
**Apo**	0.53*	0.33	3.23	0.67	3.68*		1.71
**ADP**	0.53*	0.22	3.23	0.45	3.59*	0.33	1.60
**ATP**	1.03	0.37	3.29	0.63	3.64*		1.60
**AMP-PNP**	0.53*	0.26	3.06	0.36	3.57*	0.38	1.58
**ATPγS**	0.53*	0.18	3.06	0.3	3.55*	0.50	1.64

Supplementary Table 1: Donor Fluorescence lifetime analysis for yHsp90 and hHsp90 measurements.

All the double-labeled bursts were used to estimate the fluorescence lifetimes for the donor in the presence of an acceptor (τ_1 and τ_2) and their respective fractions (f_{c1} and f_{c2}) via bi- or tri-exponential functions. The open conformation has a donor fluorescence lifetime similar to donor only lifetime. f_o is the fraction of open conformation in the data. *These lifetimes were obtained separately and were fixed in a global analysis to obtain the third lifetime component reliably.

Yeast Hsp90	**d1(Å)**	σ_1(Å)	$k_{cc\to o}$**(ms^-1^)**	**f1**	**d2 (Å)**	σ_2(Å)	$k_{o\to cc}$ **(ms^-1^)**	**f2**	**d3(Å)**	σ_D(Å)	**f3**	**d4(Å)**	σ_D(Å)	**f4**	χ^2_{red}
**Apo**	52.6	3.7		0.02	101.1	7.0		0.98							6.4
**ADP**					111.1	>10		1							5.6
**ATP**	50.8	3.6	0.65		99.9	6.9	0.14								4.5
**AMP-PNP**	56.5	3.9		0.12								89.6	6.3	0.88	6.6
**ATPγS**	51.4	3.6	1.32		95.6	6.7	0.14								9.5
Human Hsp90	**d1(Å)**	σ_1(Å)	$k_{cc\to c}$**(ms^-1^)**		**d2 (Å)**	σ_2(Å)	$k_{c\to cc}$ **(ms^-1^)**		**d3(Å)**	σ_D(Å)	**f3**				χ^2_{red}
**Apo**	50.5	3.5	0.94		81.4	5.8	0.19		38.3	2.7	0.05				3.6
**ADP**	50.5	3.5	1.72		85.0	6.6	0.76		38.6	2.7	0.05	105	3.0	0.44	1.9
**ATP**	50.5	6.0	3.04		85.7	6.0	2.6					105	7.5	0.06	6.7
**AMP-PNP**	44.1	3.0		0.03	72.3	5.1		0.86				105	7.4	0.11	3.6
**ATPγS**	43.6	3.0	1.6		75.4	5.3	1.26					83.9	5.9	0.3	5.0

Supplementary Table 2: Dynamic photon distribution analysis (PDA) of yHsp90 and hHsp90 smFRET measurements.

A dynamic PDA analysis reveals the distances for the underlying FRET populations and their respective transition rates in the ms timescales.

For yHsp90, interconversion was found between the closed compact (cc) and open (o) conformations. d1 and d2 are the respective distances with their widths (σ_1 and σ_2) for the closed compact (cc) and open (o) conformations. Their transition rates are shown as $k_{cc\to o}$ and $k_{o\to cc}$. The closed conformation is only present with AMP-PNP for yHsp90 and is given as d4. When the transitions are absent, only the fractions (f_1, f_2, f_3 and f_4) are depicted.

For hHsp90, interconversion was found between the closed compact (cc) and closed (c) conformations. d1 and d2 are the respective distances with their widths (σ_1 and σ_2) for the closed compact (cc) and closed (c) conformations. Their transition rates are shown as $k_{cc\to c}$ and $k_{c\to cc}$. A very high FRET static state is incorporated in the hHsp90 analysis for the apo and ADP conditions. An additional conformation for the hHsp90 open state is present in all the conditions except for apo and is given as d3 with their fractions as f_3.

System	Ligand	PDB ID:	Atoms	Water molecules	Salt (M)	Box dimension (Å)	Simulation time (ns)	Repeats	Total (ns)
FL WT-yHsp90	ATP	2CG9	302 154	93128	0.15	158 x 174 x 116	300	3	900
FL WT-yHsp90	*apo*	2CG9	302 104	93142	0.15	158 x 174 x 116	300	3	900
FL T13A/S109Q-yHsp90	ATP	2CG9	302 155	93127	0.15	158 x 174 x 116	300	3	900
FL T13A/S109Q-yHsp90	*apo*	2CG9	302 108	93142	0.15	158 x 174 x 116	300	3	900
NTD WT-yHsp90	ATP	2CG9	83 747	26711	0.15	83 x 114 x 93	500	3	1500
NTD WT-yHsp90	apo	2CG9	74 457	23635	0.15	93 x 81 x 105	500	3	1500
NTD T13A/S109Q-yHsp90	ATP	2CG9	83 746	26710	0.15	83 x 114 x 93	500	3	1500
NTD T13A/S109Q-yHsp90	Apo	2CG9	83 724	26718	0.15	83 x 114 x 93	500	3	1500
NTD WT-hHsp90α	ATP	7L7J	77 282	24591	0.15	111 x 90 x 82	500	3	1500
NTD WT- hHsp90α	apo	7L7J	77 248	24595	0.15	111 x 90 x 82	500	3	1500

Supplementary Table 3: List of MD simulations.

**Reliability and reproducibility checklist for molecular dynamics simulations**<br>***All boxes must be marked YES by acceptance unless an N/A option is available**	**Yes**	**N/A**	**Response (Please state where this information can be found in the text)**
**1. Convergence of simulations and analysis**			
1a. Is an evaluation presented in the text to show that the property being measured has equilibrated in the simulations (*e.g.* time-course analysis)?	☒		Fig. S19, S23, and S24 show time series for all replicas, including local convergence of the atomistic MD simulations.
1b. Then, is it described in the text how simulations are split into equilibration and production runs and how much data were analyzed from production runs?	☒		The equilibration process for the MD simulations and the length of production runs are listed in the method section (under *Atomistic molecular dynamics simulations*).
1c. Are there at least 3 simulations per simulation condition with statistical analysis?	☒		All aMD simulations were performed in pairs of 3, with time series shown for all replicas (Fig. S19, S23, and S24).
1d. Is evidence provided in the text that the simulation results presented are independent of initial configuration?	☒		Large-scale conformational changes were addressed by coarse-grained MD simulations (Fig. S22).
**2. Connection to experiments**			
2a. Are calculations provided that can connect to experiments (*e.g.* loss or gain in function from mutagenesis, binding assays, NMR chemical shifts, J-couplings, SAXS curves, interaction distances or FRET distances, structure factors, diffusion coefficients, bulk modulus and other mechanical properties, *etc.*)?	☒		We compare smFRET results with cgMD simulations, which were previously validated with SAXS data (ref. 67). The comparisons are shown and discussed in Fig. S22 and in the result section (under *Differences in the dynamics of conformational changes between the human and yeast Hps90 revealed by*

			molecular dynamics simulations).
**3. Method choice**			
3a. Is it described in the text what force field and water model are used and why?	☒		State-of-the-art atomistic force field and water model were used for the aMD and cgMD simulations (Method section under *Atomistic molecular dynamics simulations* and *Coarse-grained molecular dynamics simulations*).
3b. Do simulations contain membranes, membrane proteins, intrinsically disordered proteins, glycans, nucleic acids, polymers, or cryptic ligand binding?	☐	☒	Response not needed if **N/A**
If 3b is **YES**, are enhanced sampling methods used?	☐	☐	Response not needed if **N/A**
If enhanced sampling methods are used, are the convergence criteria clearly stated?	☐		
If 3b is **YES**, is it explained in the text why or why not enhanced sampling methods are used?	☐		
**4. Code and reproducibility**			
4a. Is a table provided describing the system setup, such as simulation box dimensions, total number of atoms, total number of water molecules, salt concentration, lipid composition (number of molecules and type)?	☒		The system setups are described in the method section (under *Atomistic molecular dynamics simulations*), as well as Table S3, which lists relevant parameters.
4b. Is it described in the text what simulation and analysis software and which versions are used?	☒		Analysis software and versions are listed in the Methods section (under *Atomistic molecular dynamics simulations*)
4c. Are initial coordinate and simulation input files and a coordinate file of the final output provided as supplementary files or in a public repository?	☐		Data provided in zenodo repository
4d. Is there custom code or custom force field parameters?	☒	☐	Custom force field parameters were used.
If **YES**, are they provided as supplementary profiles or in a public repository?	☒		Yes, standard CHARMM36m parameters are

				available in public repositories.

Supplementary Table 4: Reliability and reproducibility checklist for molecular dynamics simulations.

	Hsp90+ATP	Hsp90+ATP_37°C
**Data Set**	**WILDTYPE**	**MUTANT**
HDX reaction details	500 mM KCl<br>5 mM $MgCl_2$<br>6 mM ß-Me<br>20mM Hepes<br>pH= 7.5, 20°C	500 mM KCl<br>5 mM $MgCl_2$<br>6 mM ß-Me<br>20mM Hepes<br>pH= 7.5, 20°C
HDX time course (min)	0.167, 1, 10, 30, 120	0.167, 1, 10, 30, 120
HDX control samples	N/A	N/A
Back-exchange (mean / IQR)	N/A	
# of Peptides	306	306
Sequence coverage	97.7%	97.7%
Average peptide length / Redundancy	12.32 / 5.33	12.32 / 5.33
Replicates (biological or technical)	3 (technical)	3 (technical)
Repeatability	0.052 (average standard deviation)	0.055 (average standard deviation)
Significant differences in HDX (delta HDX > X D)	0.331 Da (99%CI)	

Supplementary Table 5: Parameters of HDX measurements.

References

1. Jussupow, A. et al. Extended conformational states dominate the Hsp90 chaperone dynamics. *J Biol Chem* **298**, 102101 (2022).
2. Daturpalli, S., Kniess, R.A., Lee, C.T. & Mayer, M.P. Large Rotation of the N-terminal Domain of Hsp90 Is Important for Interaction with Some but Not All Client Proteins. *J Mol Biol* **429**, 1406-1423 (2017).
3. Lopez, A. et al. Client binding shifts the populations of dynamic Hsp90 conformations through an allosteric network. *Sci Adv* **7**, eabl7295 (2021).
4. Mader, S.L. et al. Conformational dynamics modulate the catalytic activity of the molecular chaperone Hsp90. *Nat Commun* **11**, 1410 (2020).
